# Supplementary material for: A Systematic Review and Meta-Analysis of the Prevalence of Small Fibre Impairment in Patients with Fibromyalgia
Source: Diagnostics (Basel). 2022 May 3;12(5):1135. doi: 10.3390/diagnostics12051135 (PMC9139885; doi:10.3390/diagnostics12051135)
Supplement: Supplementary file 1 [file diagnostics-12-01135-s001.zip › diagnostics-1687368-supplementary.pdf]

diagnostics

MDPI

Systematic Review

A Systematic Review and Meta-Analysis of the Prevalence of Small Fibre Impairment in Patients with Fibromyalgia

Eleonora Galosi, Andrea Truini * and Giulia Di Stefano

Search strategies for systematic review

Database: PubMed, EMBASE, and Cochrane Library

Additional Filters:

- Language: English
- Human studies
- Text availability: full text

Table S1. Research outcomes for each combination search in terms of sorted studies and selected studies for the systematic review.

Combination search	N. of sorted studies	N. of selected studies
**Studies assessing somatic small fibres**	**209**	**43** *
Fibromyalgia AND corneal confocal microscopy	21	4
Fibromyalgia AND laser evoked potentials	26	8
Fibromyalgia AND microneurography	4	2
Fibromyalgia AND pain related evoked potentials	38	0
Fibromyalgia AND skin biopsy	120	23
Papers retrieved from second search	-	8
**Studies assessing autonomic small fibres**	**256**	**45** *
Fibromyalgia AND deep breathing	12	0
Fibromyalgia AND heart rate variability	115	29
Fibromyalgia AND laser doppler	7	3
Fibromyalgia AND quantitative sudomotor axon reflex testing	0	0
Fibromyalgia AND skin conductance	67	7
Fibromyalgia AND sympathetic skin response	19	7
Fibromyalgia AND tilt test	28	3
Fibromyalgia AND Valsalva	8	0
Papers retrieved from second search	-	0
**Total study number**	**360**	**88**
Duplicates	85	-

* Some studies assessed more than one objective test, thus the sum of the single selected studies for each combination search overcomes the total number of selected studies (i.e. 43 studies assessing somatic small fibres and 45 assessing autonomic small fibres).
